# Lens subluxation after use of a percussion massage gun: A case report

**DOI:** 10.1097/MD.0000000000031825

**Published:** 2022-12-09

**Authors:** Jiancheng Mu, Wei Fan

**Affiliations:** a Department of Ophthalmology, West China Hospital of Sichuan University, Chengdu, Sichuan Province, China.

**Keywords:** acute angle-closure glaucoma, lens subluxation, percussion massage gun, percussive therapy, trauma

## Abstract

To report the first case of lens dislocation and secondary acute angle-closure glaucoma (AACG) following use of a percussion massage gun (PMG) around the eye.

**Patient concerns:**

A 69-year-old Chinese man had been using a PMG around his right eye for 2 months in order to relieve headache. After eye pain and blurred vision for 5 days, he went to the ophthalmological emergency department. His best-corrected visual acuity at distance was counting fingers.

**Diagnosis:**

The patient was diagnosed with lens subluxation, secondary AACG and pterygium in the right eye. Cataracts were diagnosed in both eyes.

**Interventions:**

The patient underwent phacoemulsification and anterior vitrectomy. After surgery, the patient was given eye drops containing tobramycin, dexamethasone, 0.1% bromfenac sodium hydrate ophthalmic solution and Mydrin-P for 1 month.

**Outcomes:**

At 3-month follow-up, uncorrected visual acuity in the right eye was counting fingers. The outcome of optometry in the right eye was +11.50 DS/−0.50 DC * 110°, with corrected-distance visual acuity of 4/20. IOP was 20.7 mm Hg in the right eye and 15.7 mm Hg in the left. Endothelium in the right cornea showed endothelial damage. Nevertheless, the patient reported no right eye pain anymore, and he indicated that he was satisfied with his situation.

**Lessons:**

Caregivers, sports professionals and the general public should be aware of the dangers of PMGs and the need to use them appropriately and safely, for example during self-massage and rehabilitation therapy. In particular, we recommend not using PMGs above the neck, which should be clearly indicated in instruction manuals.

## 1. Introduction

Percussive therapy has gained popularity in the therapeutic and athletic communities in recent years. Various manufacturers market percussion devices for self-massage or massage by a therapist. Such devices are able to vibrate at different frequencies up to 53 Hz.^[1]^ Several attachment heads can be fixed to the device depending on whether the target tissue is soft or bony, such that the device sends strong and rapidly pulsating strokes deep into the tissue, analogous to a small jackhammer.^[2]^ Percussive massage treatment, especially of deep tissue, can reduce pain, lactate buildup, the Golgi reflex and muscle spasms, while increasing blood and lymphatic flow as well as normalizing scar tissue.^[3]^ In this way, percussive massage may enhance recovery after injury and enhance physical performance.^[2]^ However, we are unaware of studies on the indications, contraindications, or potential adverse effects of percussive massage. Using percussive massage guns (PMG) around the eye increases the risk of blunt trauma to that organ. If the zonule fibers are damaged, lens dislocation or subluxation may occur. Here we report a case of lens subluxation and AACG after repeated use of a PMG around the eye.

## 2. Case presentation

A 69-year-old Chinese man presented to the Emergency Department at West China Hospital of Sichuan University complaining of eye pain and decreased visual acuity in the right eye for the previous 5 days (Fig. 3), accompanied by photophobia, tearing, headache, nausea and vomiting. During that time, he visited a local hospital, where intraocular pressure (IOP) in the left eye was 14.0 mm Hg, but the pressure could not be measured in the right eye. To lower the pressure in the right eye, the patient received a prescription for eye drops containing 1% brinzolamide, 2% carteolol hydrochloride and 0.5% pilocarpine nitrate. Pain in the right eye was slightly better after 1 day of using the eye drops, and the IOP in the right eye was 58.0 mm Hg. The local hospital suggested that the patient visit a major hospital for further treatment.

**Figure 1.**
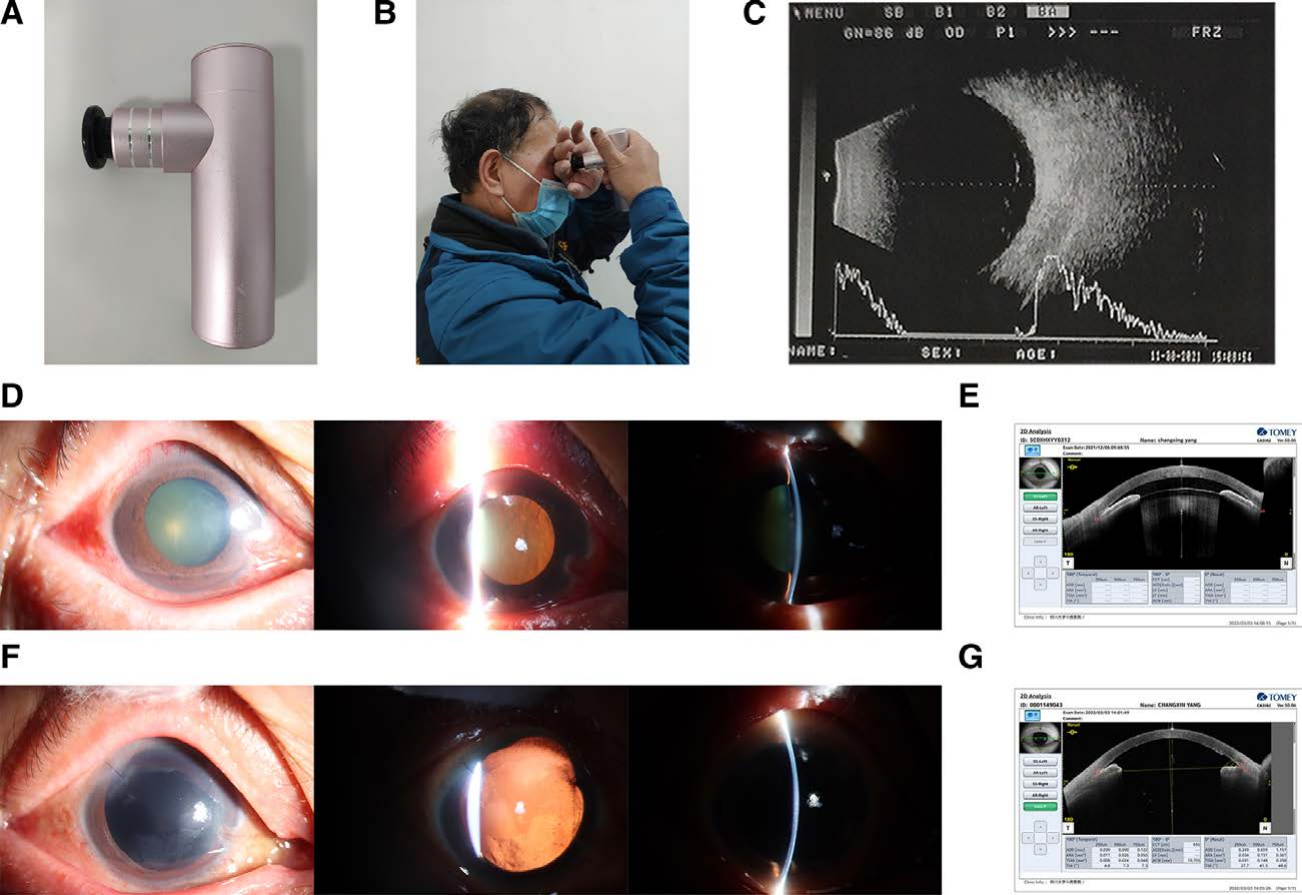
(A) Photograph of the massage gun. (B) The patient demonstrates how he used the massage percussion gun on his right eye. (C) B-scan ultrasonography of the right eye before surgery. (D) Slit-lamp microscope examination of the right eye before surgery. (E) Anterior segment optical coherence tomography of the right eye before surgery. (F) Slit-lamp microscope examination of the right eye 3 months after surgery. (G) AS-OCT of the right eye before surgery. AS-OCT = anterior segment optical coherence tomography.

**Figure 2.**
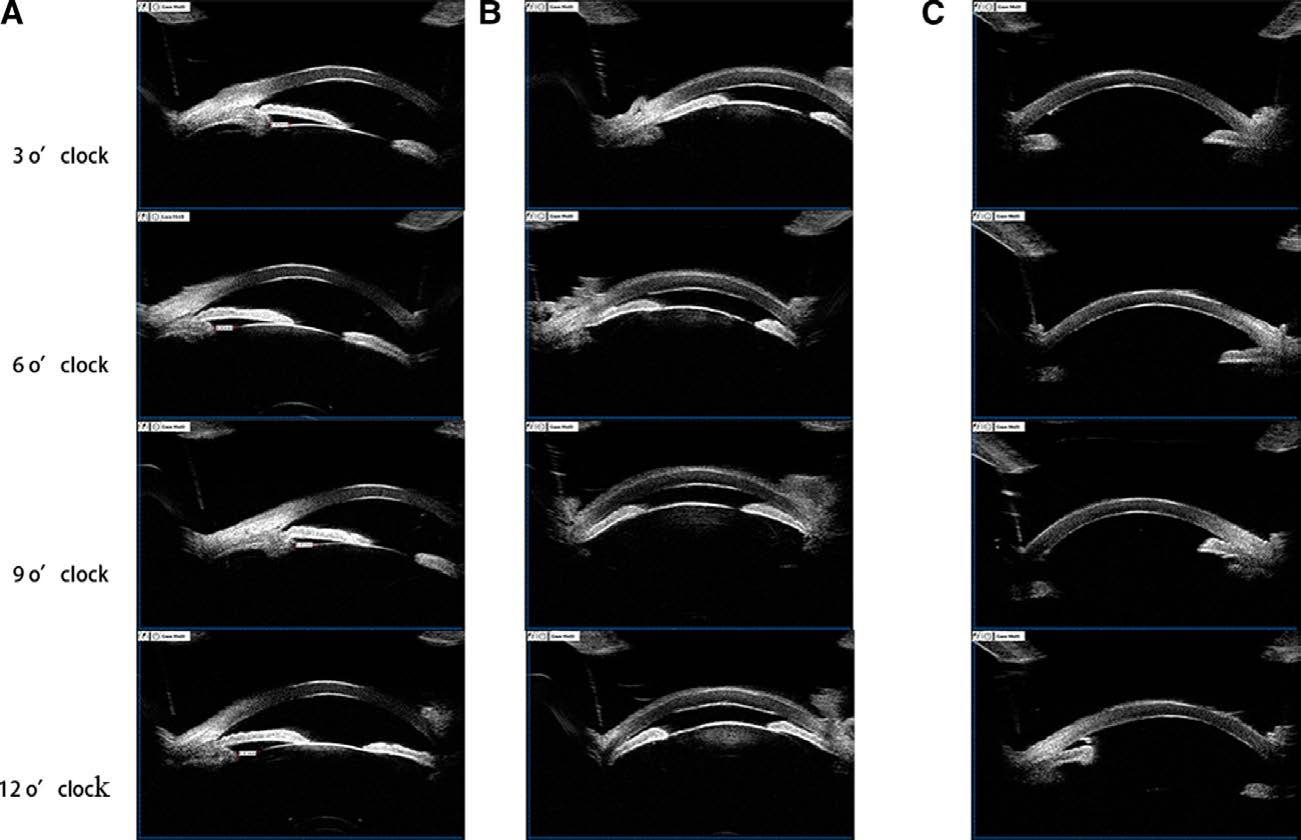
(A-B) UBM of (A) the left eye and (B) the right eye, before surgery. (C) UBM of the right eye 3 months after surgery. UBM = ultrasound biomicroscopy.

**Figure 3.**
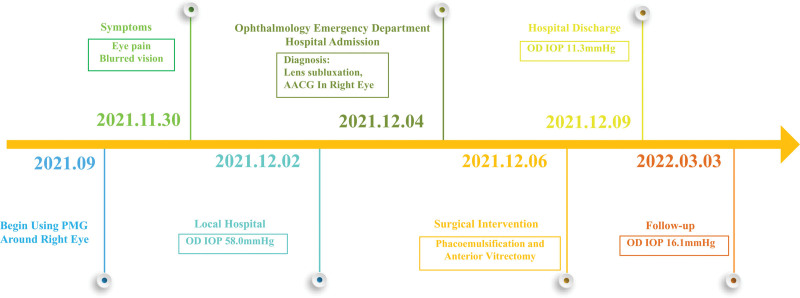
Case report timeline.

Eight hours before admission to our Emergency Department, eye pain in the right eye worsened. The patient denied a history of “trauma” and reported a history of colon polypectomy and cholecystectomy. He mentioned that he had been self-performing percussive massage around his right eye using a commercial PMG (Fig. 1A). He had been conducting such massage for about 1 minute per night for the previous 2 months in order to relieve headache. He would put 1 hand over the right eye and position the head of the massage gun on the hand (Fig. 1B). He had used the massage gun on the evening before onset of pain in the right eye that led him to come to our Emergency Department.

On admission, visual acuity in the right eye was counting fingers, which did not improve with correction. Visual acuity in the left eye was 20/20. IOP in the right eye was high (28.0 mm Hg), whereas IOP was 12.9 mm Hg in the left eye. Slit-lamp microscopy of the right eye showed mild edema in the eyelid and hyperemic conjunctiva (Fig. 1D). The right eye also showed thickened triangular layers of conjunctiva extending from the nasal edge to the cornea, edematous cornea, and a folded corneal endothelium. The anterior chamber was shallow centrally and peripherally, and the iris and lens had shifted forward. The diameter of the right pupil was 6 mm, and it did not constrict under light. After movements of the right eye, lens tremor was observed. The right lens was cataractous, and the retina showed normal attachment. The left eye showed no obvious abnormalities, except a mildly opaque lens.

Ultrasound biomicroscopy (UBM) showed that in the left eye no obvious abnormality (Fig. 2A), but in right eye, the anterior surface of the iris was in contact with the corneal endothelium and anterior chamber angle was closed, moreover the lens was away from its original position and moved forward, which could be clearly seen in the image of 9 O’clock (Fig. 2B). Anterior segment optical coherence tomography (AS-OCT) of the right eye showed the anterior chamber was shallow (Fig. 1E). The left eye showed no obvious abnormality with AS-OCT. B-scan ultrasonography did not detect vitreous body and retinal abnormalities in right eye (Fig. 1C) and left eye.

Based on these findings and the patient’s history and symptoms, he was diagnosed with lens subluxation, secondary AACG and pterygium in the right eye, as well as cataracts in both eyes.

The patient was given the following to control IOP in the right eye: methazolamide tablets, intravenous mannitol (250 mL, 20%), and eye drops containing carteolol hydrochloride, brinzolamide, brimonidine tartrate and pilocarpine nitrate. After 2 days of this treatment, IOP remained high in the right eye. Then the patient underwent phacoemulsification and anterior vitrectomy (Fig. 3). On the day after surgery, visual acuity in the right eye was hand motion, and IOP was 23.7 mm Hg in the right eye and 12.3 mm Hg in the left eye. The patient reported partial relief of right eye pain. On day 3 after surgery, IOP was 11.3 mm Hg in the right eye and 14.0 mm Hg in the left, and the patient was discharged.

After discharge, the patient was given eye drops containing tobramycin and dexamethasone (Alcon Laboratories, Fort Worth, TX), which he was instructed to take 4 times a day for the first week, 3 times a day for the second week, twice a day for the third week, and once a day for the fourth week. He was also given 0.1% bromfenac sodium ophthalmic solution (Bronuck, Senju Pharmaceutical Co., Ltd., Osaka, Japan), which he was instructed to take twice a day for 1 month. Finally, he was given Mydrin-P (tropicamide and phenylephrine; Santen Pharmaceutical Co., Ltd., Osaka, Japan), which he was told to take twice a day for 1 month.

At 3-month follow-up (Fig. 3), uncorrected visual acuity was counting fingers in the right eye and 20/20 in the left. Corrected-distance visual acuity was 4/20 (+11.50 SD/–0.50CD * 110°) in the right eye, and 20/20 (+1.50SD/–0.50CD * 120°) in the left. IOP was 16.1 mm Hg in the right eye and 13.3 mm Hg in the left. The right eye showed good recovery and substantially better condition than at admission, based on ocular photography (Fig. 1F) and UBM (Fig. 2C), in which we could see the anterior chamber depth was normal and the anterior chamber angle was open. AS-OCT of the right eye showed corneal endothelial damage (Fig. 1G), and corneal endothelial cells could not be counted in the right eye, suggesting a substantial damage of corneal endothelial cells. Nevertheless, the patient reported no right eye pain and indicated that he was satisfied with the treatment. Once the condition of his cornea in right eye improves, he will be considered for intraocular lens implantation.

## 3. Discussion

To the best of our knowledge, this is the first report of lens subluxation and secondary AACG associated with the use of a PMG. Our patient had been applying the massage gun to his right eye for 2 months, and although the head of the PMG did not directly contact the eyeball, his hand conducted the vibrations from the gun into the eye.

Our case illustrates that lens tremor after rapid eye movement and UBM examination may help detect occult lens dislocation. Such dislocation may be associated with glaucoma. The lens of the right eye in our patient showed anterior luxation toward the chamber, resulting in pupillary block, shallow chamber and closed angle. The dislocated lens can cause pupillary block by preventing the flow of aqueous humor from the posterior chamber through the pupil into the anterior chamber, where it normally exits the eye in the iridocorneal angle. The close contact between iris and lens at the pupil increases pressure of the aqueous humor in the posterior chamber, forcing the peripheral iris forward over the trabecular meshwork, and ultimately closing the anterior chamber angle. Detection of a shallow anterior chamber by split lamp examination may indicate angle-closure glaucoma or high risk of this condition. A closed angle can be confirmed using AS-OCT and UBM. Anterior lens luxation requires emergency treatment, since prolonged contact of the lens with the corneal endothelium can permanently decompensate the cornea, and the high IOP can damage the optic nerve.

Our patient did not have Marfan syndrome, homocysteinuria, or spherophakia, all of which increase risk of lens dislocation.^[4,5]^ Similarly, his history of cataracts probably did not contribute to lens dislocation, leaving percussion massage as the most likely explanation. One of the most common causes of lens subluxation or luxation is blunt external trauma.^[6]^ Lens dislocation caused by blunt injury is usually associated with glaucoma, which can cause permanent vision loss. We attribute our patient’s lens subluxation to chronic vibration, which presumably loosened and weakened the zonule fibers. Our case suggests that not only violent trauma but also long-term vibration can cause lens subluxation.

Traumatic dislocation of the lens and associated glaucoma should be diagnosed and treated promptly in order to avoid corneal decompensation and damage to the optic nerve. How to treat the AACG depends on whether the glaucoma involves pupillary block or not. If pupillary block is not yet present, the glaucoma can be treated with intravenous or oral acetazolamide, coupled with analgesia and antiemetics. If pupillary block is present, the most common treatment is laser peripheral iridotomy, in which a laser burns a small hole through the iris in order to restore aqueous flow into the anterior chamber.^[7]^ If, however, the glaucoma is induced by lens dislocation, lensectomy may be required as soon as possible. It may also be beneficial when an opacified lens impairs vision or when anterior lens dislocation has brought the lens and cornea in contact with each other. Depending on the location of the dislocated lens, lensectomy may involve a planned extracapsular cataract extraction or phacoemulsification, intracapsular cataract extraction, or pars plana lensectomy with vitrectomy.^[8]^ Our patient was treated successfully with phacoemulsification and anterior vitrectomy.

Our case highlights the need for consumer education about the appropriate use of massage percussion guns. These devices should not be used above the neck, where there are fewer soft tissues such as muscle or fat: since controlling the vibrational force is difficult, the risk of damage to delicate tissues such as eyes is high. The risk of injury may be even higher in individuals with underlying health conditions.

## Acknowledgments

We are grateful to the patient and to Ming Zhang, MD of West China Hospital, for helpful suggestions.

## Author contributions

**Conceptualization**: Jiancheng Mu, Wei Fan.

**Data curation**: Jiancheng Mu.

**Investigation**: Jiancheng Mu.

**Methodology**: Jiancheng Mu, Wei Fan.

**Writing—original draft**: Jiancheng Mu.

**Writing—review & editing**: Jiancheng Mu, Wei Fan.
